# Impact of injection dose, post-reconstruction filtering, and collimator choice on image quality of myocardial perfusion SPECT using cadmium-zinc telluride detectors in the rat

**DOI:** 10.1186/s40658-015-0111-6

**Published:** 2015-03-11

**Authors:** Asuka Mizutani, Ichiro Matsunari, Masato Kobayashi, Kodai Nishi, Wataru Fujita, Yoshiharu Miyazaki, Stephan G Nekolla, Keiichi Kawai

**Affiliations:** School of Health Sciences, College of Medical, Pharmaceutical and Health Sciences, Kanazawa University, 5-11-80 Kodatsuno, Kanazawa, 920-0942 Japan; Clinical Research Department, The Medical and Pharmacological Research Center Foundation, Wo 32, Inoyama, Hakui, Ishikawa 925-0613 Japan; Wellness Promotion Science Center, College of Medical, Pharmaceutical and Health Sciences, Kanazawa University, 5-11-80 Kodatsuno, Kanazawa, 920-0942 Japan; Department of Radioisotope Medicine, Atomic Bomb Disease Institute, Nagasaki University, 1-12-4, Sakamoto, Nagasaki, 852-8523 Japan; Department of Cardiology, Kanazawa Medical University, 1-1 Daigaku, Uchinada, Ishikawa, 920-0265 Japan; Department of Nuclear Medicine, Technical University Munich, Ismaninger Str., Munich, 81675 Germany

**Keywords:** Cadmium-zinc telluride, Collimator, Injection dose, Post-reconstruction filtering, Rat, SPECT

## Abstract

**Background:**

The aims of this study were (1) to evaluate the impact of injection dose, post-reconstruction filtering, and collimator choice on image quality of myocardial perfusion single-photon emission computed tomography (SPECT) using cadmium-zinc telluride (CZT) detectors and (2) to determine how these factors affect measured infarct size in the *in vivo* rat.

**Methods:**

Twenty-four healthy and eight myocardial infarct (MI) rats underwent myocardial perfusion SPECT imaging after injection of various doses (25 to 200 MBq) of ^99m^Tc-tetrofosmin using a standard (STD) five-pinhole collimator and high-sensitivity (HS) five-pinhole collimator. Image quality score, contrast-to-noise ratio, sharpness index, coefficient of variation (CV), and measured defect size were assessed as measures of image quality.

**Results:**

The image quality score increased and CV decreased as a function of injection dose. The contrast-to-noise ratio increased and sharpness index decreased as a function of Gaussian kernel size. When STD and HS were compared, HS tended to show higher image quality score and lower CV than STD. The use of post-reconstruction filter significantly improved image quality score and lessened CV. The reproducibility of defect size measurements, as assessed by intraclass correlation coefficients (ICC), between the collimators was poor-to-moderate (ICC = −0.31~0.57) with low (25 MBq) injection dose and with no or light (1.5-mm kernel size) filtering, whereas it was good-to-excellent (ICC = 0.75~0.97) with high (200 MBq) dose or low dose with heavy (2.5-mm kernel size) filtering. The filtering-related reproducibility was poor (ICC = −0.18~0.17) for STD with low injection dose, whereas it was good-to-excellent (ICC = 0.79~0.89) for HS. Furthermore, there was a filtering-related underestimation of defect size particularly with the use of heavy smoothing.

**Conclusions:**

Appropriate imaging setting is important to obtain high quality images and thereby reliable measurements using a preclinical myocardial SPECT in the rat. When only a low injection dose (25 MBq) is allowed, we would recommend to use HS with light (1.5-mm kernel size) filtering.

## Background

Preclinical molecular imaging such as single-photon emission computed tomography (SPECT) and positron emission tomography (PET) using small animals is increasingly being recognized as an important tool for cardiovascular research [1,2]. In particular, SPECT equipped with multi-pinhole collimators enables small-animal imaging with high spatial resolution and reasonable sensitivity [3-5]. In this context, there are a number of studies that used preclinical SPECT to assess myocardial perfusion in rodents [6-10]. It is important to note, however, that high image quality is essential for reliable measurements of myocardial images using such a preclinical SPECT system. Furthermore, the image quality is likely to be affected by many factors such as injection dose [11], reconstruction settings [5,11], post-reconstruction filtering [11], and collimator choice [12]. In clinical myocardial perfusion imaging studies, there is an increasing body of evidence that a newer generation cadmium-zinc telluride (CZT)-based SPECT camera equipped with multi-pinhole collimators provides high image quality and thereby high diagnostic performance even when injection dose is low [13,14]. To date, however, there is no data available in the literature that has focused on the impact of imaging settings on image quality of myocardial perfusion SPECT using CZT detectors in rodents. Such data would be necessary for reliable SPECT measurements before a biomedical question can be addressed.

The aims of this study were (1) to evaluate the impact of injection dose, post-reconstruction filtering, and collimator choice on image quality of myocardial perfusion SPECT using CZT detectors and (2) to determine how these factors affect measured infarct size in the *in vivo* rat.

## Methods

### System description

All acquisitions were performed using the eXplore speCZT (GE Healthcare, Milwaukee, WI, USA) preclinical SPECT system. Its characteristics and performance have been reported in detail previously [5]. Briefly, this system has a stationary detector with interchangeable rotating collimators. The detector consists of ten CZT-based detector panels surrounding the field of view (FOV). Each CZT detector panel consists of 32 × 32 arrays with a pixel size of 2.46 × 2.46 mm.

We used two collimators dedicated for rat imaging; a standard (STD) five-pinhole collimator and custom-made high-sensitivity (HS) five-pinhole collimator, which was available on request from TriFoil Imaging, Inc. (Northridge, CA, USA). The characteristics of STD have been described previously [5]. In brief, it has five pinholes of 1 mm in diameter and bore of 89 mm in diameter with radius of rotation/focal length of 50/70 mm. The characteristics of HS are virtually the same as those of STD except that the pinhole size is increased to 1.5 mm in diameter aiming at high sensitivity. The sensitivities of HS and STD were 321.0 and 138.5 cps/MBq, respectively. The axial and transaxial spatial resolutions of HS were 1.47 mm/2.68 mm and 1.45 mm/2.64 mm in full-width at half-maximum (FWHM)/full-width at tenth-maximum (FWTM), whereas those of STD were 1.11 mm/2.02 mm and 1.20 mm/2.18 mm in FWHM/FWTM, respectively.

### Reconstruction settings

All the images were reconstructed using maximum-likelihood expectation maximization (MLEM) with 50 iterations with and without resolution recovery function, based on the recommendation in our previous study [5]. The voxel size was set to 0.5 × 0.5 × 0.5 mm. Neither attenuation nor scatter correction was performed. Reconstructed transaxial images were transferred to a workstation for further processing and data analysis.

### Animal preparation

All animal procedures were approved by the Institutional Committee at the Medical and Pharmacological Research Center Foundation and were conducted in compliance with the American Heart Association requirements regarding the use of research animals. A total of 32 male Wister rats (8 to 11 weeks old, 250 to 320 g) (Japan SLC Inc., Hamamatsu, Japan) were housed for 1 week under a 12-h light/12-h dark cycle with free access to food and water. The rats were divided into two groups: the healthy rats (*n* = 24) and myocardial infarct (MI) rats (*n* = 8). The healthy rats received no intervention. The MI rats were anesthetized with an intraperitoneal injection of pentobarbital (30 mg/kg) and maintained with 2% isoflurane and then incubated and ventilated using a small animal ventilator (SN-480-7 × 2T, Shinano, Tokyo, Japan). After left thoracotomy and exposure of the heart, a 7-0 polypropylene suture on a small curved needle was passed through the myocardium beneath the proximal portion of the left coronary artery (LCA), and both ends of the suture were passed through a small vinyl tube to make a snare. The suture material was pulled tightly against the vinyl tube to occlude the LCA. Myocardial ischemia was confirmed by ST-segment elevation on the electrocardiogram and regional cyanosis of the myocardial surface. The LCA was occluded for 30 min, followed by reperfusion by release of the snare.

### Image acquisition

Each rat was imaged twice; one with STD and the other with HS in a random order. On day 1, the healthy rats were injected with 25 MBq (*n* = 6), 50 MBq (*n* = 6), 100 MBq (*n* = 6) or 200 MBq (*n* = 6) of ^99m^Tc-tetrofosmin via tail vein. Approximately 10 min later, the rats were scanned for 24 min under anesthesia with 1.5% to 2.0% isoflurane using imaging parameters of 20 s/view and 72 views/pinhole (a total of 360 views) in 1° increments. On the following day (day 2), the rats received the same dose of ^99m^Tc-tetrofosmin as day 1 and were scanned again using the same imaging parameters except that the collimator was different from that used on day 1. One or two weeks after the surgery, the MI rats were also imaged twice in the same manner as the healthy rats except that injection dose was 25 MBq (*n* = 4) or 200 MBq (*n* = 4) of ^99m^Tc-tetrofosmin.

### Post-reconstruction processing and image analysis

Image reorientation, display, post-reconstruction processing, and data analysis were performed using MunichHeart, which has been used in both clinical and preclinical studies [15-17], or PMOD software (PMOD Technologies Ltd., Zurich, Switzerland), where appropriate. The transaxial images were reoriented into short, horizontal, and vertical long-axis slices for image quality assessment. Post-reconstruction smoothing filtering was applied using a Gaussian filter with 1.5- or 2.5-mm kernel size. By visual inspection of displayed tomographic data, image quality was assessed by two readers, who were unaware of collimator type, injection dose, and post-reconstruction filter settings. Because image quality assessment was performed only in the healthy rats, the readers knew that all the scans were supposed to be normal. Each SPECT image data set was assigned as 4: excellent, 3: good, 2: fair, or 1: poor. The disagreements in image quality assignment were resolved by consensus. The intra- and inter-observer agreements of image quality rating as measured by kappa-statistic using JMP10 (SAS Institute Inc., Cary, NC, USA) were 0.869 and 0.832, respectively.

**Figure 1:**
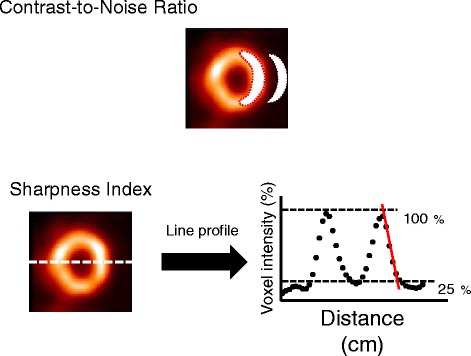
**Examples of determination of regions of interest for calculation of contrast-to-noise ratio (upper) and sharpness index (lower).** For contrast-to-noise ratio calculation, myocardial and background regions of interest (white) were drawn on a midventricular short-axis image. For sharpness index calculation, the maximal slope of epicardial border of lateral wall was determined from a line-profile curve on a midventricular short-axis image.

For quantitative analysis, we tested four parameters: (1) contrast-to-noise ratio as a measure of image contrast that may not be due to noise fluctuations [18]; (2) sharpness index as a measure of image sharpness reflecting spatial resolution [18]; (3) coefficient of variation (CV) of activity distribution as a measure of myocardial activity distribution heterogeneity; and (4) myocardial perfusion defect size. Contrast-to noise ratio and sharpness index were determined in a manner as previously described [18]. In brief, mean myocardial counts and corresponding SD were determined on a ring-shaped region of interest encompassing the endocardial and epicardial borders on the midventricular short-axis slice (Figure 1). Mean background counts and corresponding SD were determined on a half-moon-shaped region of interest on the background. Then, contrast-to noise ratio was obtained using the following formula:

$$ \left({\mathrm{Mean}}_{\mathrm{myo}} - {\mathrm{Mean}}_{\mathrm{BG}}\right)/\mathrm{SQRT}\left\{{\left({\mathrm{SD}}_{\mathrm{myo}}\right)}^2 + {\left({\mathrm{SD}}_{\mathrm{BG}}\right)}^2\right\}, $$

where Mean_myo_ is mean myocardial counts; Mean_BG_, mean background counts; SD_myo_, standard deviation (SD) of myocardial counts; and SD_BG_, SD of background counts.

Sharpness index was determined on the horizontal profile of the midventricular short-axis slice (Figure 1). This index was computed as the maximal slope of the decrease in myocardial counts (cm^−1^) on the epicardial border of the lateral wall.

The CV and defect size were determined using a polar map approach. The polar maps were normalized to the mean of six connected sectors showing the highest overall uptake in the left ventricular myocardium. The CV of activity distribution over the entire left ventricular myocardium (a total of 460 sectors) was calculated as a quantitative measure of heterogeneity. Defect size was quantified using a cutoff threshold of 60% of the reference sectors and was expressed as a percentage of the left ventricular myocardium (%LV).

### Statistical analysis

Data were expressed as mean ± standard deviation (SD). Statistical analysis was performed using JMP10, GraphPad Prism6 (GraphPad Software, Inc., San Diego, CA, USA) or SPSS21 (IBM Japan Inc., Tokyo, Japan), where appropriate. Unpaired mean values were compared using Wilcoxon or Krukal-Wallis test. The comparison of paired mean values was performed using Wilcoxon signed-rank or Friedman test. In this study, we preferred to use non-parametric methods because it is robust irrespective of sample distribution. Defect size measurement reproducibility was assessed by intraclass correlation coefficient (ICC) and Bland-Altman plots. A *P* value <0.05 was considered significant.

## Results

### Healthy rats

**Figure 2:**
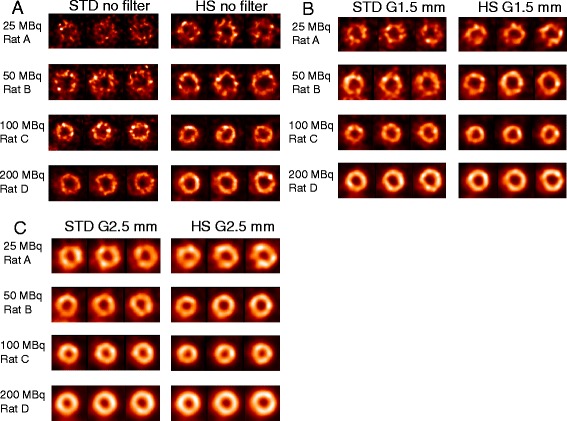
**Consecutive short-axis slices of myocardial perfusion SPECT with various injection doses (range: 25 to 200 MBq).** In the healthy rats without post-reconstruction filtering **(A)**, with Gaussian filter of 1.5-mm kernel size **(B)**, and with that of 2.5-mm **(C)**. Each rat was imaged twice; one with the standard (STD) pinhole collimator (left) and the other with the high-sensitivity (HS) pinhole collimator (right). However, different rats were presented at different doses.

**Figure 3:**
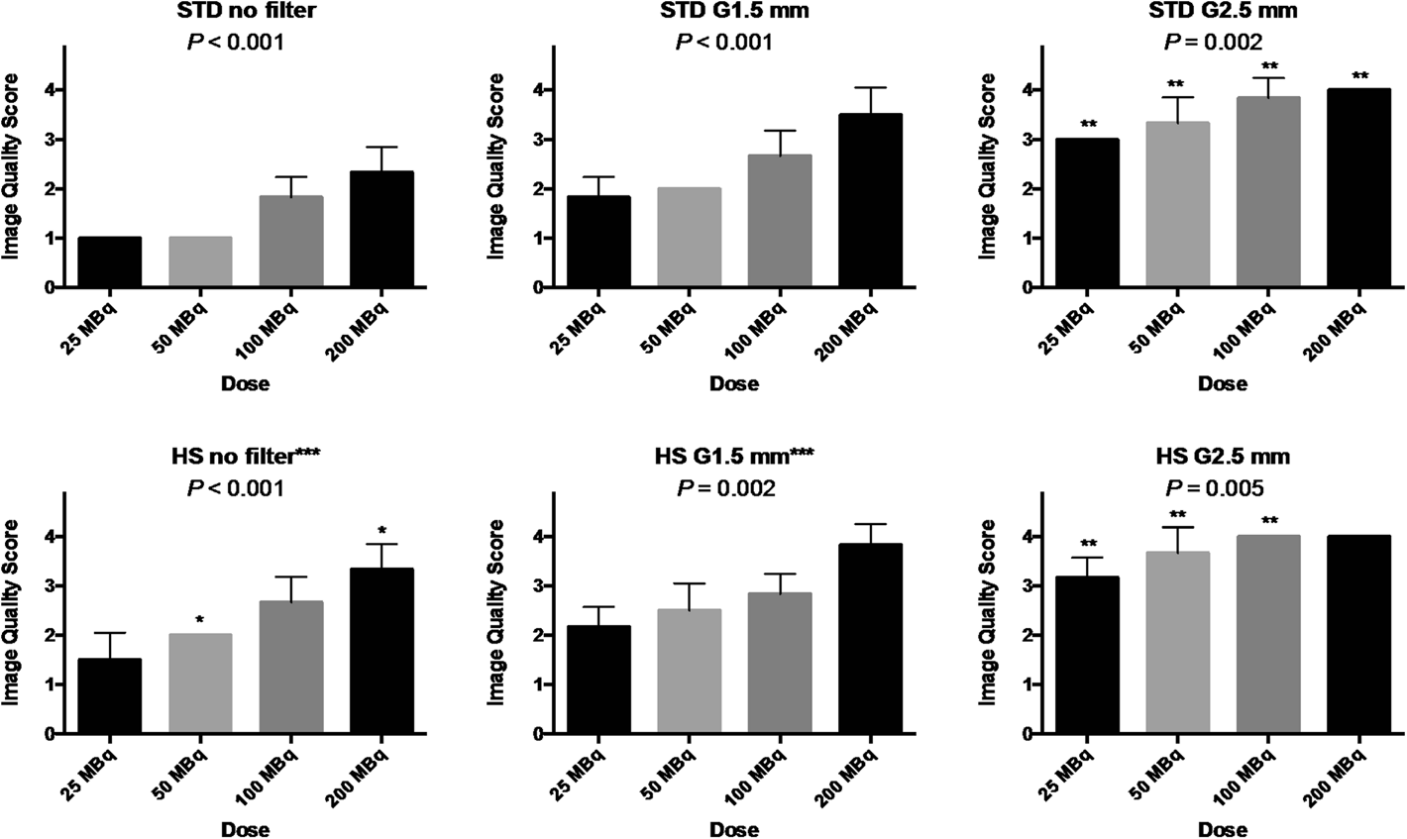
**Bar charts showing the image quality scores in the healthy rats as a function of injection dose.** Upper left: the chart with the standard (STD) pinhole collimator without post-reconstruction filter. Upper middle: the chart with STD with Gaussian filter of 1.5-mm kernel size (G1.5 mm). Upper right: the chart with STD with Gaussian filter of 2.5-mm kernel size (G2.5 mm). Lower left: the chart with the high-sensitivity (HS) pinhole collimator without post-reconstruction filter. Lower left: the chart with the STD with post-reconstruction filter. Lower right: the chart with the HS with post-reconstruction filter. The *P* value shown is based on Kruskal-Wallis test. Error bar indicates standard deviation; the bar without error bar, standard deviation of 0. * indicates *P* < 0.05 between STD and HS graphs; **P < 0.05 among no filter, G1.5 and G2.5 mm graphs; and ***P < 0.05 between STD and HS graphs using all the 24 rats as a whole.

Representative images of healthy rats with various injection doses (range: 25 to 200 MBq) using either STD or HS with and without post-reconstruction filtering are illustrated in Figure 2. Without post-reconstruction filtering, the image quality was poor when the injection dose was low (e.g., 25 MBq) particularly with STD, which improved as a function of injection dose. The use of post-reconstruction filter significantly improved image quality and homogeneity of the images as a function of Gaussian kernel size. This was confirmed by summary results of image quality assessment as shown in Figure 3. When STD and HS were compared, the image quality of HS tended to be higher than STD, which was statistically significant at the injection dose of 50 and 200 MBq without post-reconstruction filtering. However, the advantage of HS was lost when a heavy (2.5-mm kernel size) smoothing was applied.

**Figure 4:**
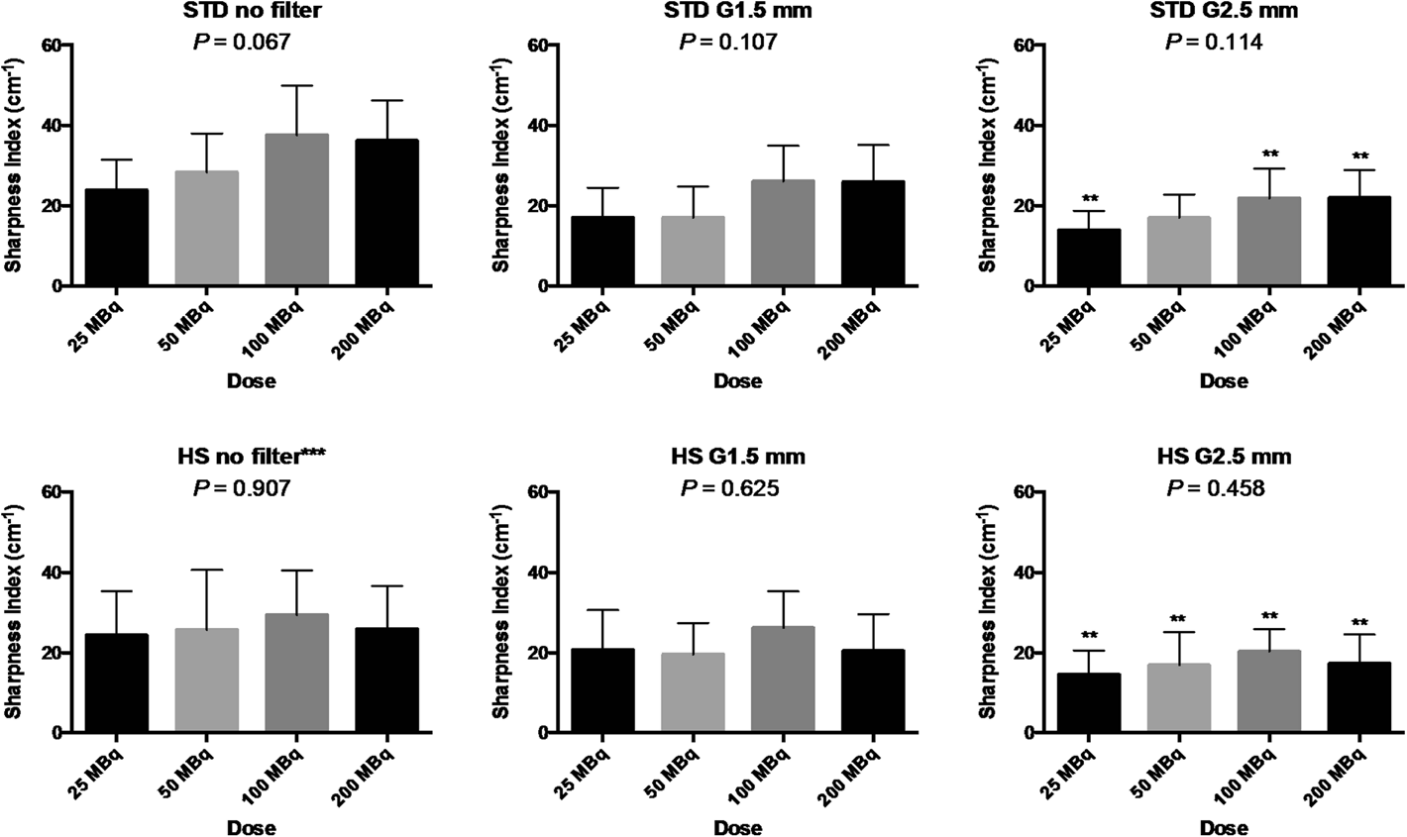
**Bar charts showing the contrast-to-noise ratios in the healthy rats as a function of injection dose.** Upper left: the chart with the standard (STD) pinhole collimator without post-reconstruction filter. Upper middle: the chart with STD with Gaussian filter of 1.5-mm kernel size (G1.5 mm). Upper right: the chart with STD with Gaussian filter of 2.5-mm kernel size (G2.5 mm). Lower left: the chart with the high-sensitivity (HS) pinhole collimator without post-reconstruction filter. Lower left: the chart with the STD with post-reconstruction filter. Lower right: the chart with the HS with post-reconstruction filter. The *P* value shown is based on Kruskal-Wallis test. Error bar indicates standard deviation. * indicates *P* < 0.05 between STD and HS graphs; **P < 0.05 among no filter, G1.5 and G2.5 mm graphs; and ***P < 0.05 between STD and HS graphs using all the 24 rats as a whole.

**Figure 5:**
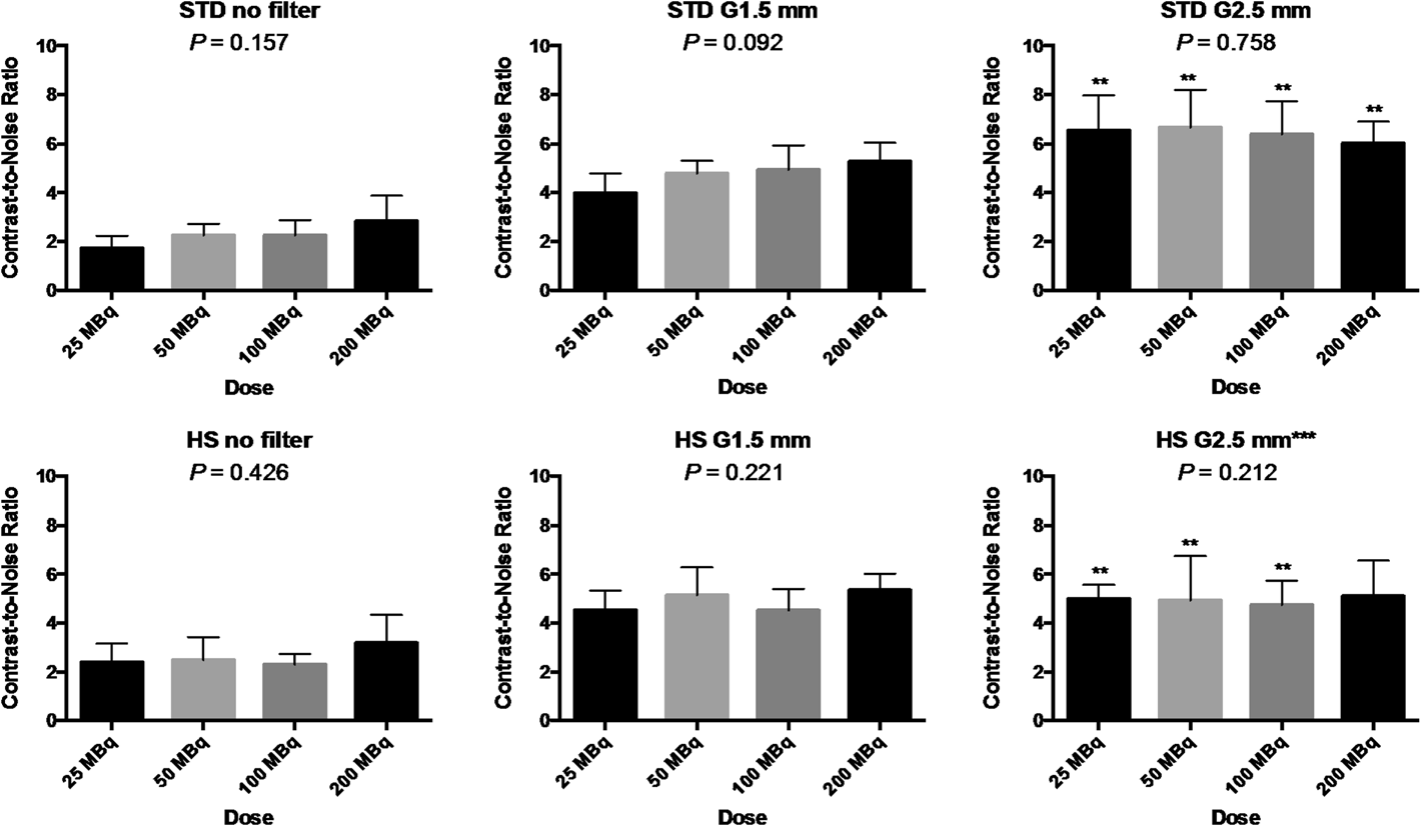
**Bar charts showing the sharpness indices in the healthy rats as a function of injection dose.** Upper left: the chart with the standard (STD) pinhole collimator without post-reconstruction filter. Upper middle: the chart with STD with Gaussian filter of 1.5-mm kernel size (G1.5 mm). Upper right: the chart with STD with Gaussian filter of 2.5-mm kernel size (G2.5 mm). Lower left: the chart with the high-sensitivity (HS) pinhole collimator without post-reconstruction filter. Lower left: the chart with the STD with post-reconstruction filter. Lower right: the chart with the HS with post-reconstruction filter. The *P* value shown is based on Kruskal-Wallis test. Error bar indicates standard deviation. * indicates *P* < 0.05 between STD and HS graphs; ** *P* < 0.05 among no filter, G1.5 and G2.5 mm graphs; and ***P < 0.05 between STD and HS graphs using all the 24 rats as a whole.

The results of contrast-to-noise ratio and sharpness index are summarized in Figures 4 and 5. The contrast-to-noise ratio generally increased and sharpness index generally decreased as a function of Gaussian kernel size in both STD and HS. Although there was a trend toward increase in the contrast-to-noise ratio or sharpness index by increasing dose with certain settings such as those seen in STD, increasing dose did not significantly alter these measures. When the STD and HS were compared using all the 24 rats as a whole, the contrast-to-noise ratio was not different between the collimators with no (2.3 ± 0.8 for STD vs 2.6 ± 0.9 for HS, NS) or light (1.5-mm kernel size) post-reconstruction filtering (4.7 ± 0.9 for STD vs 4.9 ± 0.9 for HS, NS) but was lower for HS when heavy (2.5-mm kernel size) post-reconstruction filtering was applied (6.4 ± 1.2 for STD vs 4.9 ± 1.2 for HS, *P* < 0.05). The sharpness index was lower for HS without post-reconstruction filtering (31.6 ± 11.0 cm^−1^ for STD vs 26.4 ± 11.4 cm^−1^ for HS, *P* < 0.05), but there were no significant differences between STD and HS when post-reconstruction filtering was applied (1.5-mm kernel size: 21.5 ± 9.1 cm^−1^ for STD vs 21.7 ± 8.9 cm^−1^ for HS, NS, and 2.5-mm kernel size: 18.7 ± 6.9 cm^−1^ for STD vs 17.2 ± 6.7 cm^−1^ for HS, NS).

**Figure 6:**
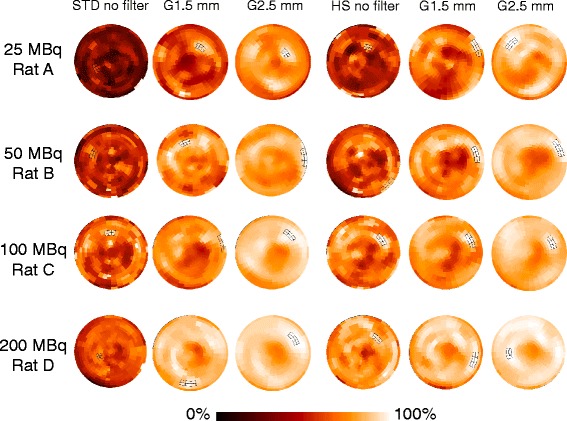
**Polar maps of myocardial perfusion SPECT with various injection doses (range: 25 to 200 MBq).** In the same healthy rats illustrated in Figure 2. Each rat was imaged twice; one with the standard (STD) pinhole collimator (left three panels) and the other with the high-sensitivity (HS) pinhole collimator (right three panels) with or without post-reconstruction filtering. G1.5 mm indicates Gaussian filter of 1.5-mm kernel size; G2.5 mm, Gaussian filter of 2.5-mm kernel size.

**Figure 7:**
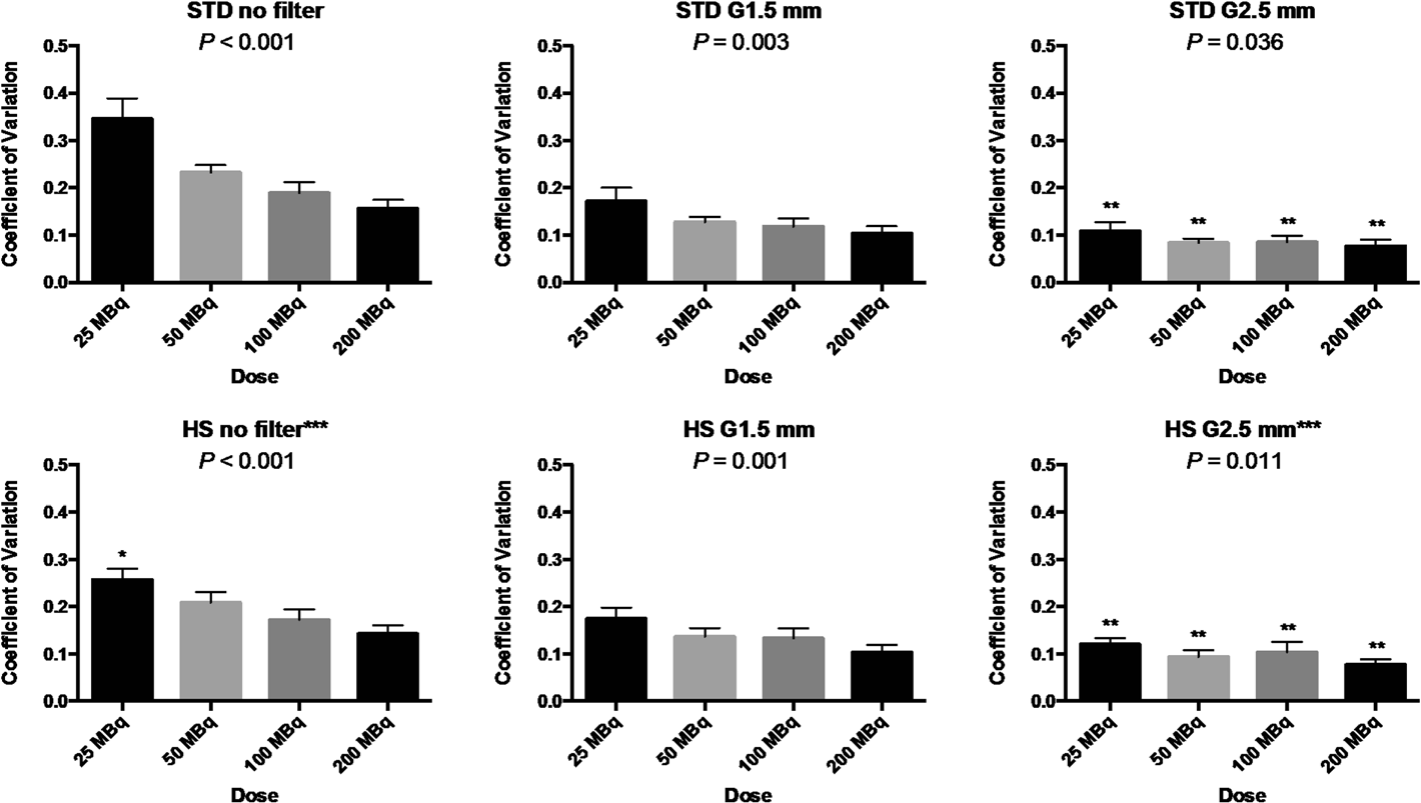
**Bar charts.** Showing the coefficient of variation of left ventricular myocardial activity distribution in the healthy rats as a function of injection dose. Upper left: the chart with the standard (STD) pinhole collimator without post-reconstruction filter. Upper middle: the chart with STD with Gaussian filter of 1.5-mm kernel size (G1.5 mm). Upper right: the chart with STD with Gaussian filter of 2.5-mm kernel size (G2.5 mm). Lower left: the chart with the high-sensitivity (HS) pinhole collimator without post-reconstruction filter. Lower left: the chart with the STD with post-reconstruction filter. Lower right: the chart with the HS with post-reconstruction filter. The *P* value shown is based on Kruskal-Wallis test. Error bar indicates standard deviation. * indicates *P* < 0.05 between STD and HS graphs; **P < 0.05 among no filter, G1.5 and G2.5 mm graphs; and ***P < 0.05 between STD and HS graphs using all the 24 rats as a whole.

Polar maps of the same healthy rats illustrated in Figure 2 are shown in Figure 6. By visual inspection, homogeneity of activity distribution improved by increasing injection dose, the use of HS instead of STD, and the use of post-reconstruction filter. CV as a quantitative measure of activity distribution heterogeneity decreased as a function of injection dose (Figure 7). The use of post-reconstruction filter significantly lessened CV for both STD and HS. When STD and HS were compared, HS showed a significantly lower mean CV than STD at the injection dose of 25 MBq without post-reconstruction filtering. The use of post-reconstruction filter significantly reduced CV values at all the injection doses tested. The effect of post-reconstruction filter as expressed by the difference between non-filtered and filtered (2.5-mm kernel size) CV values was larger at 25 MBq than that at 200 MBq for both STD (0.23 ± 0.04 at 25 MBq vs 0.10 ± 0.02 at 200 MBq, *P* < 0.01) and HS (0.15 ± 0.03 at 25 MBq vs 0.08 ± 0.02 at 200 MBq, *P* < 0.01).

### Myocardial infarct rats

**Figure 8:**
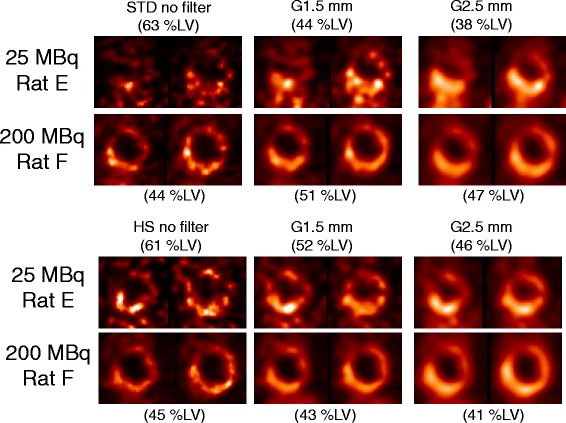
**Consecutive short-axis slices of myocardial perfusion SPECT.** With low (25 MBq) and high (200 MBq) injection doses in the rats with myocardial infarction. Each rat was imaged twice; one with the standard (STD) pinhole collimator (upper two panels) and the other with the high-sensitivity (HS) pinhole collimator (lower panels) with or without post-reconstruction filtering. The number in parentheses is the defect sizes, expressed as percentage of the left ventricular myocardium (%LV), measured on polar maps. G1.5 mm indicates Gaussian filter of 1.5-mm kernel size; G2.5 mm, Gaussian filter of 2.5-mm kernel size.

**Figure 9:**
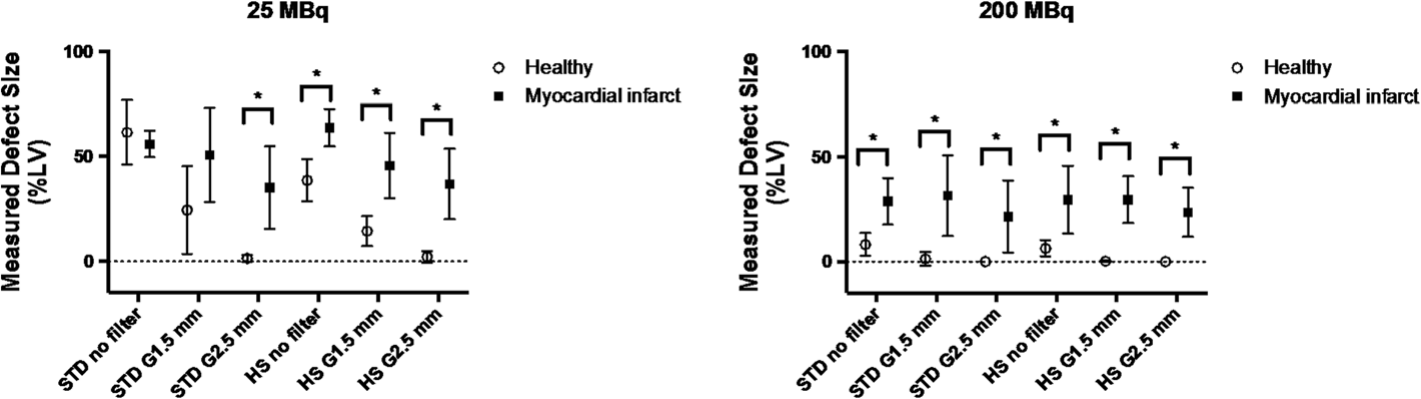
**Interleaved symbol plots comparing the measured defect size expressed as percentage of left ventricular myocardium (%LV).** With low (25 MBq) (left) or high (200 MBq) (right) injection dose between the healthy and myocardial infarct rats. Error bar indicates standard deviation; STD, the standard pinhole collimator; HS, the high-sensitivity pinhole collimator; G1.5 mm, Gaussian filter of 1.5-mm kernel size; G2.5 mm, Gaussian filter of 2.5-mm kernel size. *P < 0.05 by Wilcoxon test.

**Figure 10:**
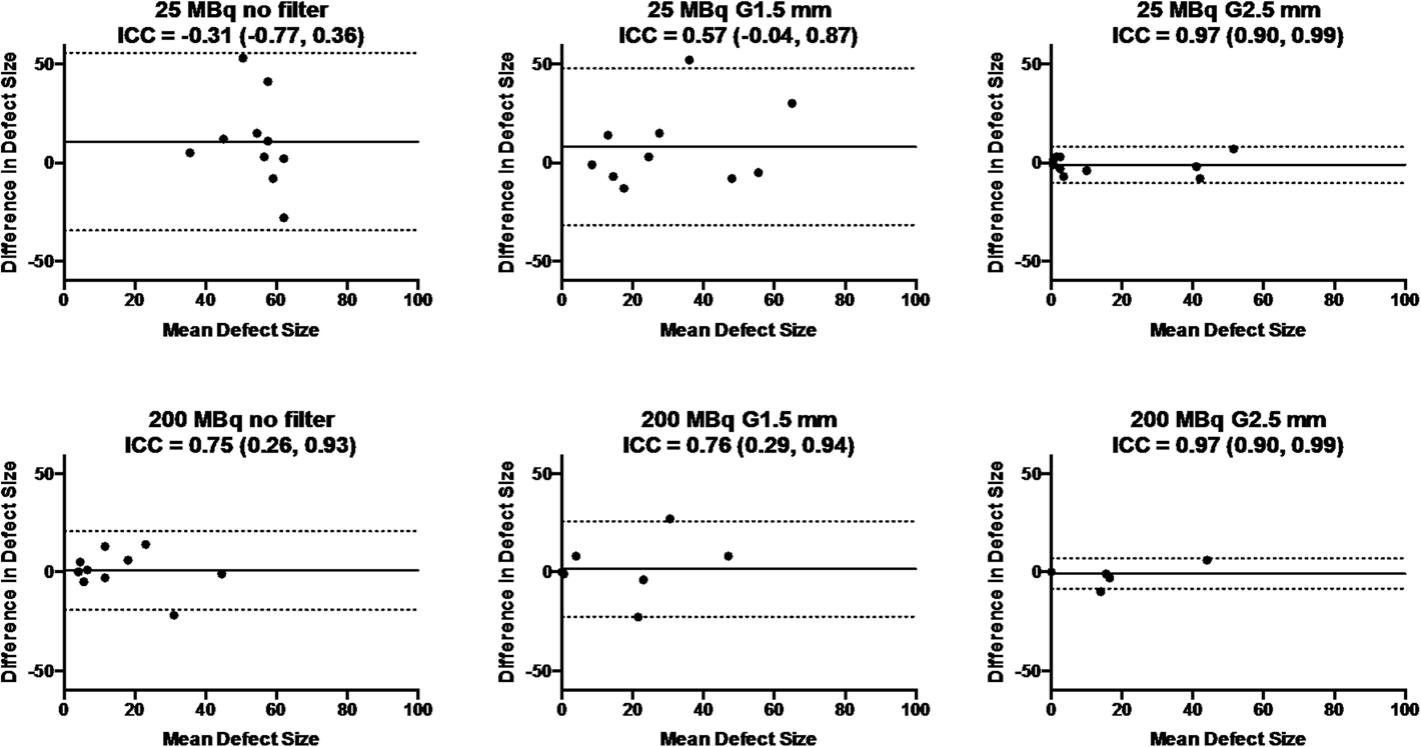
**Bland-Altman plots and intra-class correlation coefficients (ICC).** With 95% confidence intervals in the parentheses of defect size measurement reproducibility between the standard (STD) and high-sensitivity (HS) pinhole collimators. The results for low (25 MBq) injection dose are shown in upper panels; those for high (200 MBq) injection dose in lower panels. Solid lines indicate mean difference; dotted lines, 95% limits of agreement.

**Figure 11:**
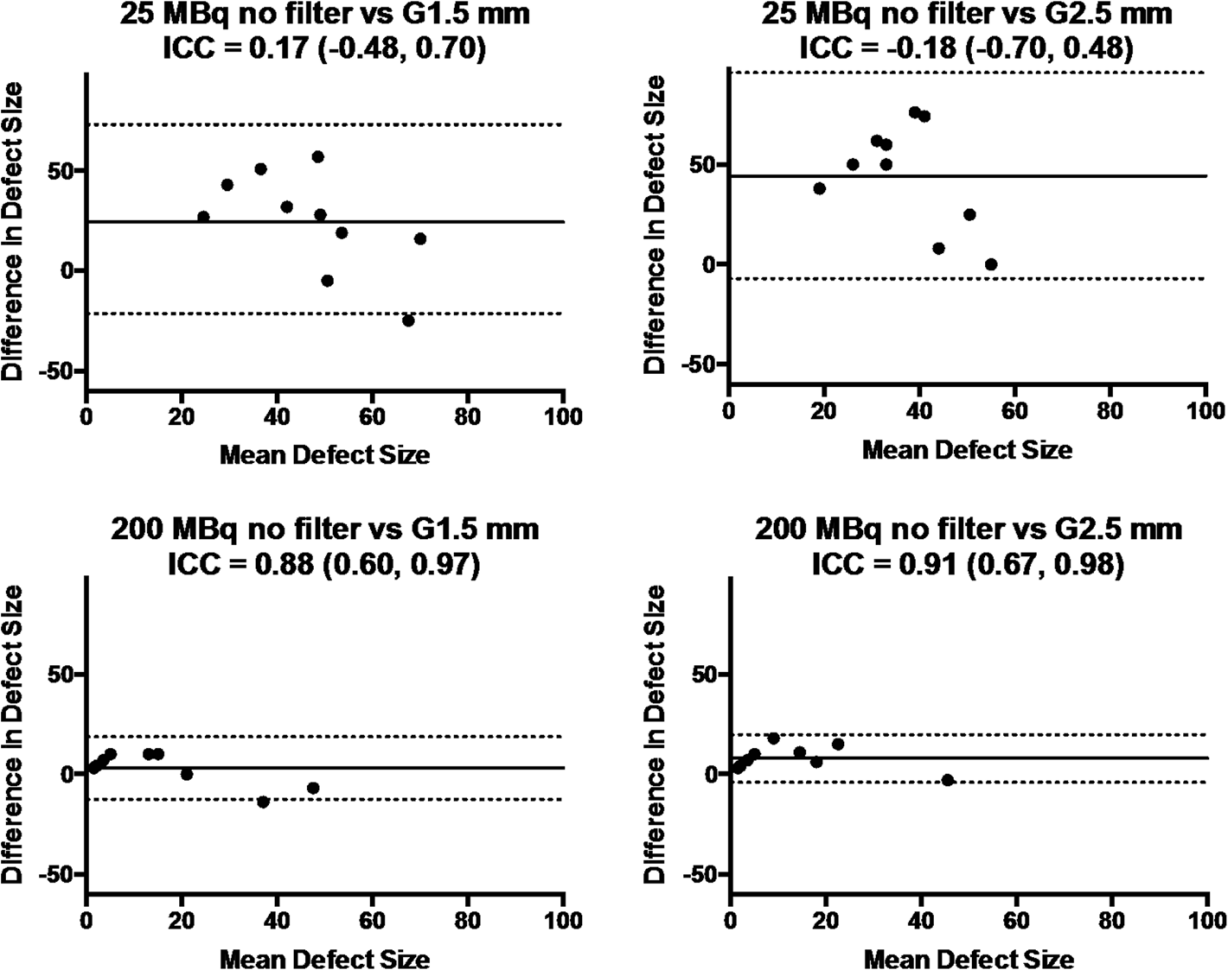
**Bland-Altman plots and intra-class correlation coefficients (ICC).** With 95% confidence intervals in the parentheses of defect size measurement reproducibility between no and light (1.5-mm kernel size) or heavy (2.5-mm kernel size) filtering for the standard (STD) pinhole collimator. The results for low (25 MBq) injection dose are shown in upper panels; those for high (200 MBq) injection dose in lower panels. Solid lines indicate mean difference; dotted lines, 95% limits of agreement; G1.5 mm, Gaussian filter of 1.5-mm kernel size; G2.5 mm, Gaussian filter of 2.5-mm kernel size.

**Figure 12:**
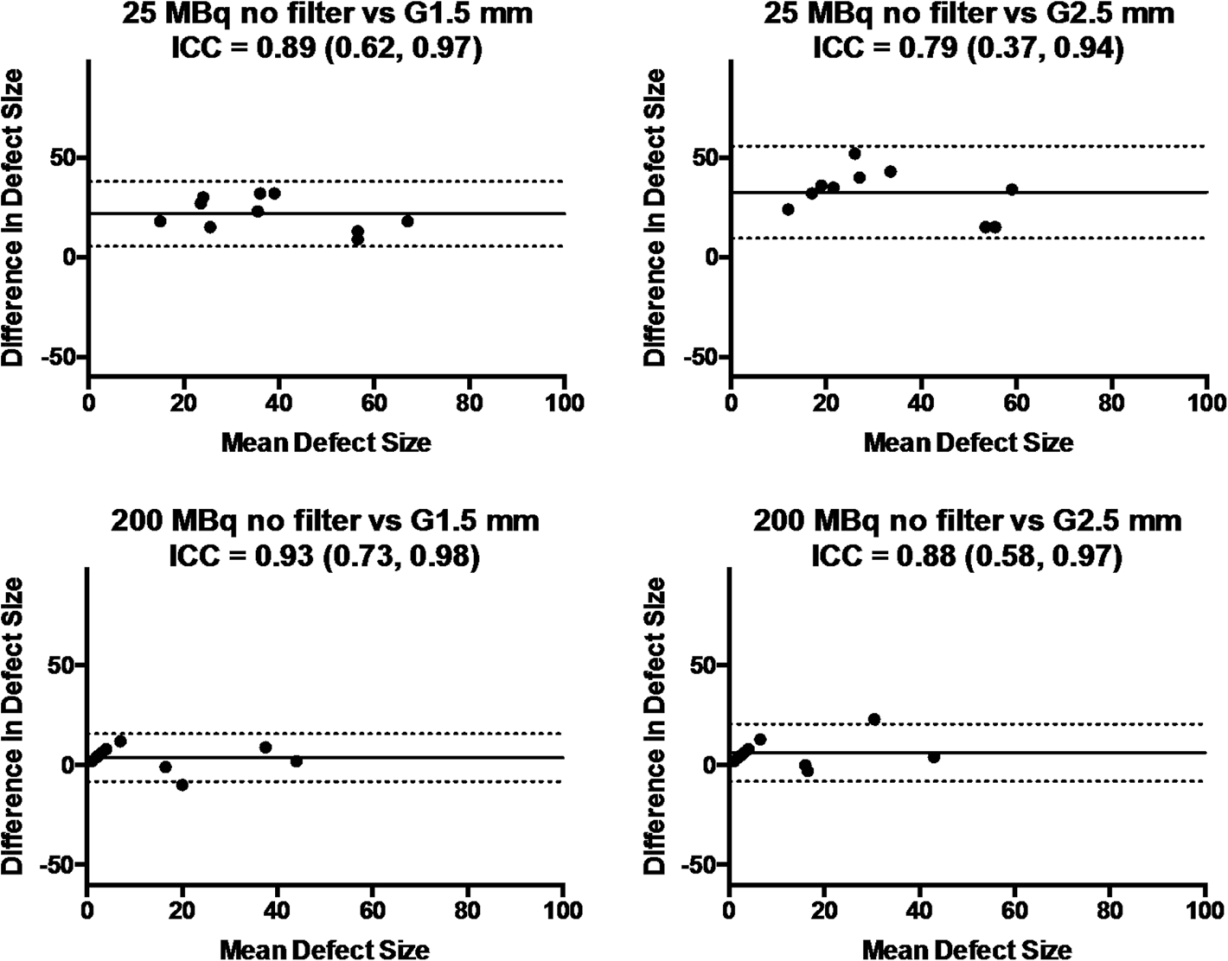
**Bland-Altman plots and intra-class correlation coefficients (ICC).** With 95% confidence intervals in the parentheses of defect size measurement reproducibility between no and light (1.5-mm kernel size) or heavy (2.5-mm kernel size) filtering for the high-sensitivity (HS) pinhole collimator. The results for low (25 MBq) injection dose are shown in upper panels; those for high (200 MBq) injection dose in lower panels. Solid lines indicate mean difference; dotted lines, 95% limits of agreement; G1.5 mm, Gaussian filter of 1.5-mm kernel size; G2.5 mm, Gaussian filter of 2.5-mm kernel size.

Representative images of MI rats with low (25 MBq) and high (200 MBq) injection doses are illustrated in Figure 8. By visual inspection, myocardial perfusion defect was difficult to identify on the 25 MBq images due to poor image quality particularly with STD, which became visible when the post-reconstruction filter was applied. On the 200 MBq images, myocardial perfusion defect was visible with either STD or HS regardless of the use of post-reconstruction filter. By quantitative analysis, the measured defect sizes were similar between the healthy and MI rats at 25-MBq injection dose with STD with no or light (1.5-mm kernel size) post-reconstruction filter (Figure 9) despite the fact that the healthy rats should not have any defects. With the use of HS or heavy (2.5-mm kernel size) post-reconstruction filter, the measured defect size in the infarct rats was larger than that in the healthy rats. When the injection dose was 200 MBq, the MI rats showed a significantly larger defect size than the healthy rats regardless of collimator choice or the use of post-reconstruction filter. Reproducibility of defect size measurements between the collimators was assessed using Bland-Altman plots and intraclass correlation coefficients (Figure 10). The reproducibility of defect size measurements was poor-to-moderate (ICC = −0.31~0.57) with low (25 MBq) injection dose and with no or light (1.5-mm kernel size) filtering, whereas it was good-to-excellent (ICC = 0.75~0.97) with high (200 MBq) dose or low (25 MBq) dose with heavy (2.5-mm kernel size) filtering. Finally, the effect of post-reconstruction filtering on reproducibility of defect size measurements was assessed for STD and HS in Figures 11 and 12, respectively. For STD, the reproducibility was poor (ICC = −0.18~0.17) with low (25 MBq) injection dose between no filter and either light (1.5-mm kernel size) or heavy (2.5-mm kernel size) filtering. Furthermore, a systemic bias toward smaller defect size with filtering was observed as a function of Gaussian kernel size. When the injection dose was high (200 MBq), the reproducibility was good-to-excellent (ICC = 0.88~0.91). There was no remarkable systemic bias toward larger or smaller defect size due to filtering. For HS, the reproducibility was good-to-excellent (ICC = 0.79~0.89) with low (25 MBq) injection dose between no filter and either light (1.5-mm kernel size) or heavy (2.5-mm kernel size) filtering. Like the results of STD, a systemic bias toward smaller defect size with filtering was observed, although the magnitude of bias appeared to be smaller than those observed for STD. When the injection dose was high (200 MBq), the reproducibility was good-to-excellent (ICC = 0.88~0.93) without a remarkable systemic bias toward larger or smaller defect size due to filtering.

## Discussion

Clinical CZT-based SPECT has successfully been applied in myocardial perfusion imaging studies, which enables shorter acquisition time and lower injection dose as compared to those of conventional NaI-based SPECT cameras mainly because of its high image quality and high contrast [13,14]. This study was aimed to determine the relationships between image quality and imaging settings in small animals such as the rat. The major findings of this study were that (1) the image quality score increased and CV decreased as a function of injection dose in both STD and HS; (2) the contrast-to-noise ratio generally increased and sharpness index generally decreased as a function of Gaussian kernel size in both STD and HS; (3) when STD and HS were compared, HS tended to show higher image quality score and lower CV than STD in the healthy rats; (4) the use of post-reconstruction filter significantly improved image quality score and lessened CV in the healthy rats; (5) the effect of post-reconstruction filter as expressed by the difference between non-filtered and filtered CV values was larger at 25 MBq than that at 200 MBq for both STD and HS; (6) the measured defect sizes were similar between the healthy and MI rats at 25-MBq injection dose with STD but were significantly different with the use of HS, post-reconstruction filter, or at the high injection dose; (7) the reproducibility of defect size measurements was poor-to-moderate with low (25 MBq) injection dose and with no or light filtering, whereas it was good-to-excellent with high (200 MBq) dose or low (25 MBq) dose with heavy filtering; and (8) the filtering-related reproducibility was poor (ICC = −0.18~0.17) for STD with low injection dose, whereas it was good-to-excellent (ICC = 0.79~0.89) for HS. Furthermore, there was a filtering-related underestimation of defect size particularly with the use of heavy (2.5-mm kernel size) smoothing.

### Injection dose

In prior myocardial perfusion SPECT studies using ^99m^Tc-labeled compounds in rats [7,8,19-23], injection dose varied considerably from study to study (37 to 555 MBq). This injection dose range in rats would be equivalent to 7,400 to 111,000 MBq in humans, assuming the body weights of 300 g for rats and 60 kg for humans. As expected, our data showed that the image quality can be unacceptably poor when injection dose is low but improved as a function of injection dose, which is supported by the results of image quality analysis as well as CV as a measure of heterogeneity. This indicates that injection dose is certainly an important factor to determine image quality and measurement reliability.

### Post-reconstruction filtering

Post-reconstruction filter is used to reduce noise and improve image quality. In a phantom study evaluating the imaging performance of a preclinical SPECT system, the use of post-reconstruction filter significantly improved image uniformity with some blurring effects [11]. Our results showed that the left ventricular myocardium was delineated even with the use of post-reconstruction filter, indicating that some blurring may not have a major effect on visibility of myocardial wall. Instead, the use of post-reconstruction filter significantly improved image quality and reduced CV and artifactual defect size in the healthy rats, which was especially true at low injection doses such as 25 MBq. However, our data indicate that there is a trade-off between the contrast-to-noise ratio and sharpness index when post-reconstruction filtering is applied, and therefore, its kernel size should be determined based on the balance between these measures. Furthermore, the results of comparison between the collimators indicate that the use of HS may reduce image contrast as reflected by lower contrast-to-noise ratio when a heavy smoothing is applied, which is likely due to the contamination of myocardial activity into the background regions of interest. Thus, in view of image contrast, a heavy smoothing should probably be avoided particularly for HS.

### Collimator

Multi-pinhole collimator is a key technology for recent success in preclinical SPECT systems [3]. In general, collimator design is an important determinant of spatial resolution, sensitivity, and FOV. Our results showed that HS provides better image quality score and homogeneity as reflected by lower CV than STD. However, spatial resolution as measured by sharpness index was worse for HS than STD particularly when no post-reconstruction filtering was applied, indicating that a care should be taken in selecting an appropriate collimator for imaging.

### Myocardial infarct rats

Another important finding of this study is that the measured defect sizes were similar between the healthy and MI rats at 25-MBq injection dose with STD, indicating that, when the image quality is poor due to inappropriate imaging settings, myocardial perfusion SPECT imaging is no longer able to distinguish MI from healthy rats. This is also supported by the poor reproducibility observed when injection dose was low (25 MBq) with no filtering. However, this problem can substantially be improved by changing imaging settings such as increasing injection dose, using post-reconstruction filter, and appropriate collimator choice. Thus, optimization of imaging settings is essential for reliable measurements in preclinical myocardial SPECT before a biomedical question can be addressed.

### Defect size measurement reproducibility

In view of defect size measurement reliability, the measurements should be reproducible without a significant systemic bias. Therefore, we performed reproducibility analysis of defect size measurement between the collimators and between no filtering and filtering using Bland-Altman plots and ICCs, as measures of reproducibility. When a good reproducibility was defined as an ICC of ≥0.75, this was achieved between the collimators and between no filtering and filtering with a high (200 MBq) injection dose, indicating that collimator choice and the use of post-reconstruction filtering do not significantly affect measurement reproducibility in this dose setting. With a low (25 MBq) injection dose, the good reproducibility was observed between the collimators when a heavy (2.5-mm kernel size) smoothing was applied. However, such a heavy smoothing would cause a significant underestimation of defect size as compared to no filtering setting particularly with STD as illustrated in Figures 11 and 12. More importantly, the filtering-related reproducibility was poor for STD irrespective of filter kernel size (Figure 11), indicating that the defect size measured using STD may not be reproducible in such a low-dose setting. By contrast, the filtering-related reproducibility was much better for HS as illustrated in Figure 12. However, there still remained a filtering-related underestimation of defect size particularly with the use of heavy (2.5-mm kernel size) smoothing. Therefore, one would use a light (1.5-mm kernel size) smoothing rather than a heavy (2.5-mm kernel size) one in view of measurement reproducibility.

### Recommendations for optimal imaging settings

An optimal imaging setting would be one that provides high image quality score, contrast-to-noise ratio, sharpness index, and low CV. Furthermore, the defect size measurements should be reproducible and should discriminate the MI rats from the healthy rats. However, there is a trade-off between these image quality parameters. Therefore, the imaging setting should be determined based on what is of concern. Because myocardial perfusion SPECT is often performed to measure perfusion defect size, we assumed that the optimal imaging setting would be one that can reliably distinguish MI from healthy rats. In this regard, when a high injection dose (200 MBq) is allowed, this requirement is met with high reproducibility irrespective of collimator choice and the use of post-reconstruction filtering. If we consider the adverse effect of post-reconstruction filtering (i.e., loss in sharpness index and thereby spatial resolution), one would use STD with no filtering. The choice of optimal settings becomes critical when only a low injection dose (25 MBq) is allowed. The use of STD is not recommended unless images are heavily smoothed, resulting in significant loss in spatial resolution. Alternatively, one could use HS to distinguish MI from healthy rats. In this setting, the use of light (1.5-mm kernel size) smoothing is recommended because artifactual defect in the healthy rats is still substantial without filtering as illustrated in Figure 9. Conversely, a heavy (2.5-mm kernel size) smoothing may result in loss in spatial resolution (sharpness index) and image contrast (contrast-to-noise ratio). Therefore, we would recommend to use HS with light filtering when a low injection dose is allowed.

### Limitations

There are limitations of the study to be described. First, we had to use a large number of rats to compare the effects of injection dose. The sample size could have been reduced with the use of partial reconstruction of the data in the same rats with different acquisition times. This would be possible with state-of-the-art SPECT systems such as U-SPECT II [24], which has completely stationary detector and collimator design. However, such a strategy was not possible with our SPECT camera that has stationary detector but rotating collimator design [5], where the entire scan procedure needs to be completed to obtain projection data over 360°. Faster rotations and subsampling could be an alternative. However, this was not possible because averaging projection data from repeated acquisitions was not implemented in our system. From physiological point of view, tracer uptake of the heart as well as that of adjacent organs in rodents is not constant over time as demonstrated in an experimental study [25], indicating, for example, that the 1 and 24 min acquisition data started 10 min after injection may not necessarily be the same in view of tracer kinetics of target organs. For these reasons, we applied a fixed time duration (between 10 and 34 min after injection) rather than different acquisition times to compare the effect of different doses. Alternatively, repeated acquisitions using the same animals may solve the problem. However, it means that a rat should be scanned eight times (four dose settings for each collimator), which would require at least eight imaging days and could be stressful for the rats. Because the rats used in this study were planned to be reused in other studies later to reduce the overall number of rats required, we wished to avoid to put too much stress on them. Therefore, we set a limit of two imaging days (i.e., STD and HS) per rat. Nevertheless, the use of partial reconstruction, if available, would be a more efficient way to compare the total count-image quality relationship than what was done in this study. Second, the lack of attenuation and scatter correction is another limitation of this study. Finally, our results obtained in the rat are not directly applicable to mice studies. Because mice are much smaller than rats (i.e., ten times lower body weight), optimal imaging settings such as collimator choice and injection dose for mice should be different from those for rats and therefore should be determined in further studies using collimators dedicated for mice imaging.

## Conclusions

Our data indicate that appropriate imaging setting is important to obtain high quality images and thereby reliable measurements using a preclinical myocardial SPECT in the rat. If these are not met, its measurements can be unreliable with inability to distinguish MI from healthy rats. When a high injection dose (200 MBq) is allowed, we would recommend to use STD with no filtering. When only a low injection dose (25 MBq) is allowed, we would recommend to use HS with light (1.5-mm kernel size) filtering. Such a validation and optimization of imaging settings are necessary in preclinical myocardial SPECT before a biomedical question can be addressed.
